# “It’s starting to weigh on me”: Exploring the Experiences and Support Needs of Harm Reduction Staff in Connecticut using the Social-Ecological Model

**DOI:** 10.1186/s12954-023-00898-4

**Published:** 2023-11-14

**Authors:** Katherine Hill, Katherine Dunham, Lauretta E. Grau, Robert Heimer

**Affiliations:** 1 Department of Epidemiology of Microbial Diseases, Yale School of Public Health, New Haven, CT USA; 2 Department of Social and Behavioral Sciences, Yale School of Public Health, New Haven, CT USA

**Keywords:** Harm reduction, Staffing, Burnout, Mental health, Qualitative

## Abstract

**Background:**

The experiences and perceived support needs of harm reduction workers in the USA have been understudied. While previous research has explored staff burnout and role-related stress, there is a research gap around potential supports for staff wellbeing and individual longevity in their roles. This is especially critical given the growing overdose crisis and the need for sustainable harm reduction programming. Thus, we sought to describe the experiences of harm reduction staff and identify the perceived support that could empower harm reduction staff to successfully navigate their roles.

**Methods:**

Purposive sampling methods were used to recruit harm reduction staff working in Connecticut. Seventeen semi-structured, one-on-one interviews were conducted between December 2022 and March 2023. Participants were asked about their experiences with role-related stressors and supports. Informed by the Social-Ecological Model, transcripts were coded using both inductive and deductive codes, and themes were developed using thematic analysis approaches.

**Results:**

Study participants described their experiences working in harm reduction and the numerous ways they already are or could be receiving support in their roles. These experiences were organized into eight themes according to the levels of the Social-Ecological Model. At the individual level, participants explained that support could help them navigate the variability of the physical environment, boundary setting, and self-care. Relationships between clients and co-workers were both identified as means of support at the interpersonal level, helping participants navigate difficult situations and feelings of stress. At the organizational level, study participants explained how they look to their organization to provide sufficient support by way of training, staffing, compensation, and benefits. Additionally, participants stressed the importance of having supervisors who valued their work and provided emotional support. Lastly, at the community level, participants discussed how support was needed to help them navigate complex systems while working with a stigmatized population in an often-stigmatized field.

**Conclusions:**

To best support harm reduction staff in their day-to-day roles, our findings underscore the need for support on multiple levels. Future research could explore how the provision of support to harm reduction staff impacts not only staff perceptions of support but also the success of clients accessing harm reduction services.

**Supplementary Information:**

The online version contains supplementary material available at 10.1186/s12954-023-00898-4.

## Background

Staff employed by harm reduction programs work in a variety of settings (e.g., syringe service programs, overdose prevention sites, medical clinics, substance use treatment programs, etc.) and their job titles vary accordingly (e.g., outreach worker, community health worker, peer navigator, overdose responder, etc.). Despite this variety, harm reduction staff have the common responsibility of engaging with people who use drugs (PWUD) to meet them where they are and offer strategies to reduce harms related to drug use (e.g., educating people on safer use practices, supplying sterile syringes or naloxone, and connecting clients with social or medical services). While there is a breadth of research on harm reduction interventions, most studies examine experiences and outcomes related to the clients who access these services (i.e., PWUD) rather than the individuals providing these services.

Only a few studies have described harm reduction staff experiences and, importantly, their perceived support needs. Some common stressors among the harm reduction workforce have been previously identified, such as grief, trauma stewardship, burnout, and inadequate funding and support [1]. Additionally, studies exploring the effect of the overdose crisis on those responding to overdose events, such as emergency medical technicians and other first responders, demonstrate the toll these responsibilities incur: high rates of burnout, increased workloads, and increased perceptions of helplessness [2, 3]. Furthermore, studies show that those working with stigmatized populations in healthcare settings have been found to experience stigma by association, leading to increased job stress and social isolation [4, 5].

Research has also explored the unique challenges of harm reduction workers who themselves use drugs [6–8]. Because these staff members—commonly referred to as “peer navigators'' or “peer workers”—often have deep connections to the community they serve, they are likely to face additional stress and trauma when responding to adverse events [7, 9–13]. This additional burden is associated with increased burnout and staff turnover [14]. Further, discrimination and structural barriers may prevent the formal hiring of people who are actively using drugs or create pay and benefit inequities for peer workers as compared to co-workers who are not using drugs [7, 14, 15].

Existing studies have also found that paid sick leave and “wellness checks” may be supportive for harm reduction staff’s mental health, countering role-related stressors [16]. Harm reduction organizations have also recognized that staff connectedness is critical in promoting employee wellness and improving the benefit of debriefings following adverse client events, such as overdoses, arrests, or violence [17–19].

However, the perceived support needs of harm reduction staff have been understudied, especially in the USA and beyond the context of the COVID-19 pandemic. First, most studies on harm reduction staff experiences have been conducted in Canada, where both the criminal-legal system and public opinion tend to be more supportive of harm reduction strategies than in the USA [20–23]. In addition to this variation in support, harm reduction staffs’ experiences likely differ between Canada and the USA from factors that may impact the clients of harm reduction organizations (e.g., funding for social services or access to universal healthcare coverage in Canada). Second, recent qualitative studies in the USA were conducted during the height of the COVID-19 pandemic when unprecedented changes impacted the harm reduction field (e.g., supply chain shortages, closures of physical sites, changes to syringe exchange policies) [24–26]. Understanding how to support harm reduction staff in the USA, beyond the COVID-19 pandemic and its related impacts, is imperative to the sustainability and success of such programs. Thus, we conducted a Connecticut-based qualitative study to (a) describe the experiences of harm reduction staff and (b) identify potential opportunities for support that could empower harm reduction staff to successfully navigate their roles. The state is a choice location for such a study because harm reduction staff are employed in a wide range of direct service, consulting, and research programs.

## Methods

### Recruitment procedures and study sample

Using purposive sampling methods, we contacted managers from harm reduction programs, substance use treatment programs, and harm reduction research sites in Connecticut that (1) primarily offered harm reduction services or opioid use disorder treatment access and (2) employed harm reduction-focused participant navigators or community health workers. The programs were identified by a research team member (R.H.) who has many years of experience working with the Connecticut harm reduction community. All of the programs that were contacted (*n *= 6) responded; one program chose not to participate due to management turnover. Potential participants were identified by the managers of the programs who provided the research team with their staff’s names and email addresses. The research team then contacted all identified potential participants (*n *= 22) over email to inform them of the purpose of this voluntary study and invite them to participate.

Potential participants indicated their interest in the study and availability for an interview through a brief, online screener. Participants were considered eligible if they were (a) currently employed in direct client service, (b) 18 years of age or older, and (c) English language proficient. The final sample size was determined by theoretical saturation, agreed upon by the research team and defined as the point when there were no new concepts emerging from interviews [27]. A total of 17 eligible individuals were contacted by a member of the research team (K.H.), verbal consent was obtained, and an interview time and setting that worked best for each of them was determined.

### Instrument development and data collection

Prior to development of the research instrument, the lead author (K.H.), a PhD student in epidemiology with prior training in qualitative and quantitative research methods, shadowed staff at various harm reduction sites around Connecticut, observing the work practices and daily duties of staff. After this observation period, the lead author (K.H.) and a faculty mentor (R.H.) designed an interview guide to probe key domains that were identified through this shadowing experience (i.e., role-related training, skills pertinent to harm reduction roles, working with clients, providing services, and potential frustrations) (Additional file 1: Appendix 1).

The lead author (K.H) conducted one-time, individual, semi-structured interviews with study participants between December 2022 and March 2023. Depending on participants’ comfort and preference, interviews took place in-person or over Zoom. After obtaining verbal consent from study participants, interviews were audio-recorded. In-person interviews occurred in small, private office spaces and the Zoom-based participants were asked to be in a quiet, private place at the time of their interview. On average, the interviews lasted approximately one hour (range = 40–93 min).

Given that participants were recruited from a small, tight-knit community of workers where people largely know one another, no demographic data were collected. However, participants did provide information related to their role (e.g., their current employer and title), years of experience in harm reduction, and personal experience with substance use. Participants were compensated $25 for their time and expertise. As interviews progressed, the study team met regularly to revise the interview guide and assess theoretical saturation [27]. One research team member (K.D.) transcribed and anonymized the interviews and the lead author (K.H.) proofread the transcripts for accuracy.

### Data analysis and the social ecological model

**Fig. 1:**
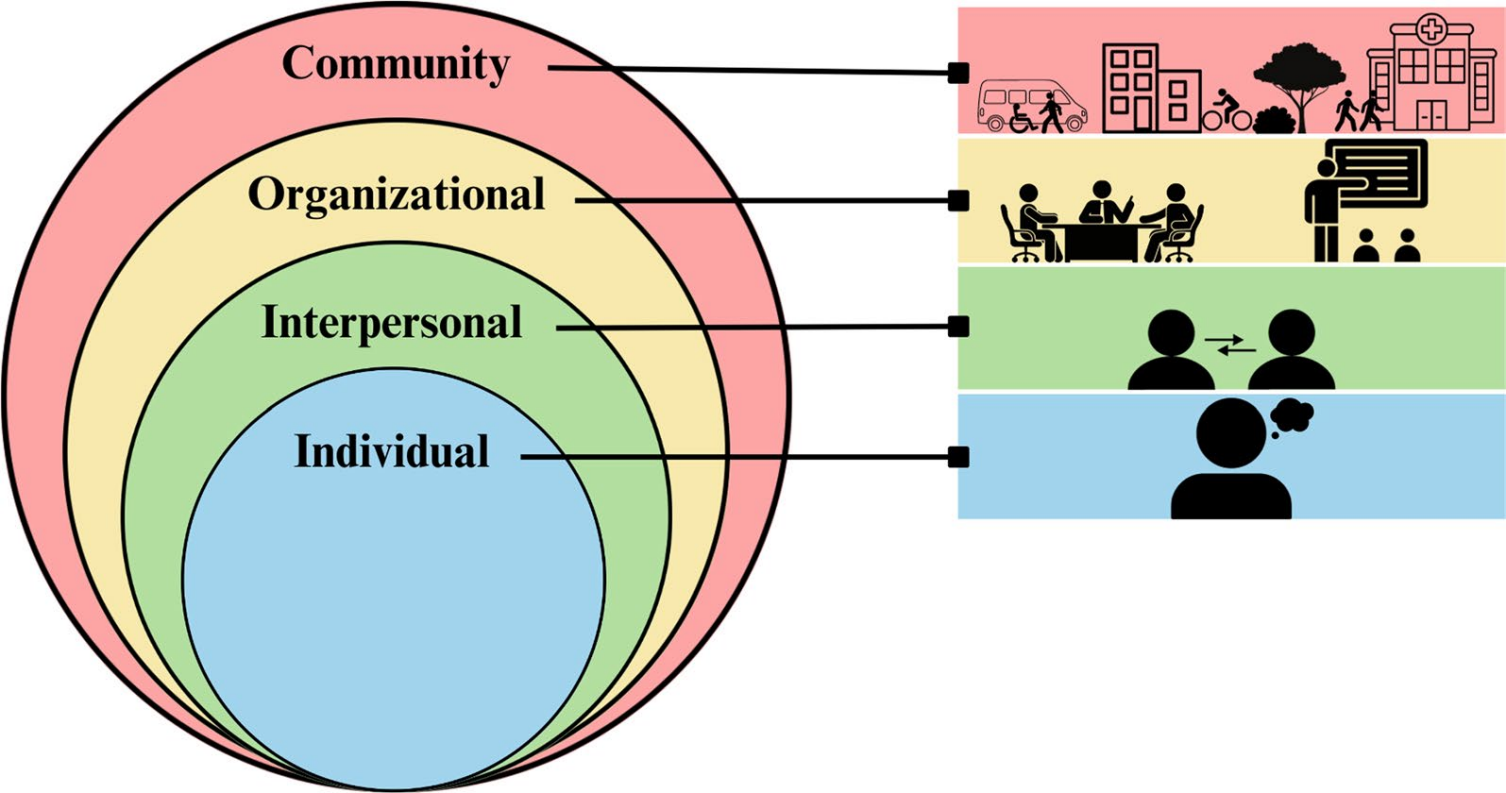
Social-Ecological model for barriers and facilitators of support for harm reduction staff

The research team analyzed the data using a thematic analysis approach [28]. After reviewing the transcripts (*N *= 17) and discussion between all members of the research team, a codebook was developed using both inductive and deductive codes. The codebook was theoretically grounded in the Social-Ecological Model (SEM), which provided a framework to inform our understanding of how support needs for harm reduction staff operate on and interact across multiple levels of influence (i.e., individual, interpersonal, organizational, community) [29, 30]. At the model’s core, the individual level focuses on intrapersonal factors such as a person’s identity, physical health, and emotional wellbeing. Moving outward, the interpersonal level explores dynamics of relationships between colleagues, friends, family, and others in their social network. The organizational level evaluates how regulations and operations of organizations (i.e., harm reduction employers) influence perceived staff support needs. The outermost layer in our adapted model is the community level which concerns collaborations between organizations and how harm reduction staff come to fit into their broader communities (Fig. 1).

The coding team (K.H., K.D.) independently coded all transcripts and then met to review their respective code assignments and reach consensus on any coding discrepancies. L.G. provided mentorship on the qualitative methods. Transcripts were entered into NVivo version 12 (QSR International) where the codes were organized for thematic analysis.

### Ethical considerations

This study was determined to be exempt from human subjects review following expedited review by the Yale University Human Research Protection Program based on the study interviews being not behavioral in nature but instead related to participants’ employment. Anonymity for study participants was promised in consent materials. At the time of the interview, verbal consent was received from all participants, and they were reminded that they could end their interview at any time, for any reason.

## Results

### Participant characteristics

The sample of 17 participants primarily served their clients in community settings as opposed to brick-and-mortar clinical settings. They held a variety of titles and worked in a range of settings and localities across Connecticut. All staff interviewed in this study were responsible for trying to connect clients to basic necessities such as housing or food, referring clients to treatment for medical care related to drug use, providing clients with safer use supplies, and educating clients on how to prevent harms associated with drug use. A few staff members had more specialized training, and thus had additional responsibilities like providing first aid or counseling. While almost 60% of participants had prior work experience in harm reduction or a related field, most participants (*n *= 11) had been in their current role for less than a year. Specific study participant characteristics, including information about the nature of participants’ employers, can be found in Table 1. Text data from study participants was collected through a mix of in-person (*n *= 12) and virtual (*n *= 5) interviews.

**Table 1:** Descriptive statistics of study participants currently employed in harm reduction roles in Connecticut (*N *= 17)

Characteristics	Frequency	Percent
*Primary employer*		
Research program	6	35.3
Mental health and substance use treatment program	7	41.2
Harm reduction service delivery program	4	23.5
*Primary title*		
Patient navigator/peer navigator/community health worker	4	23.5
Research assistant	4	23.5
Overdose responder	6	35.3
Other	3	17.7
*Primary county served*		
New Haven	6	35.3
New London	3	17.7
Hartford	1	5.9
Fairfield	2	11.8
Litchfield	5	29.4
*Time in current role*		
Under 1 year	11	64.7
1 year–5 years	3	17.7
6 + years	1	5.9
Prior job experience in harm reduction or a related field*		
Yes	10	58.8
No	7	41.2
*Personal experience with substance use*		
Yes	6	35.3
No	11	64.7

*Related fields include areas such as addiction services

### ***Theme overview***

Through thematic analysis, we explored harm reduction staff’s perceived support needs. Ultimately, we identified eight themes across four levels of the SEM: individual, interpersonal, organizational, and community levels. Table 2 provides an overview of these themes and sub-themes organized by the SEM.

**Table 2:** Themes organized by social-ecological model level

Individual level
* Theme 1* Navigating the variability of the physical environment
* Theme 2* Navigating boundary setting and self-care
* Subtheme 2.1* Feelings of guilt
* Subtheme 2.2* Impact of lived experiences
Interpersonal level
* Theme 3* Establishing and maintaining relationships with clients
* Theme 4* Establishing and maintaining relationships with fellow staff
Organization level
* Theme 5* Receiving affirmation and care from supervisors
* Theme 6* Receiving resources and benefits from the organization
* Subtheme 6.1* Training and shadowing
* Subtheme 6.2* Staffing
* Subtheme 6.3* Compensation and benefits
Community level
* Theme 7* Working with a stigmatized population in a stigmatized field
* Subtheme 7.1* Onus of public education
* Subtheme 7.2* Ideological misalignment
* Theme 8* Working within complex and inefficient systems
* Subtheme 8.1* Navigating bureaucracy
* Subtheme 8.2* Perceived helplessness

### Individual level

#### Theme 1: Navigating the variability of the physical environment

Study participants, especially those who served their clients via outreach in community settings as opposed to those who engaged with clients onsite, reported experiencing significant variability in the surrounding physical environment. For instance, many participants explained that serving clients out in the community (e.g., on a mobile van) added variability to their role and responsibilities for the day (e.g., being in areas with high foot traffic meant interacting with many non-clients who were unfamiliar to the staff, etc.). This variability consequently influenced intrapersonal factors, such as participant’s physical comfort and feelings of security. There was a range in how participants perceived their individual experiences and needs while engaging in outreach in the community, but most participants highlighted that their physical comfort could be supported through active boundary setting and building trust with clients. For instance, one participant explained how having a strong relationship with their clients meant that staff of their program were able to feel safe even in unpredictable environments:

*We work in dangerous areas. One time I was working night shift and we pulled up and the client was like "Get out of here, it's hot on the block." … Then we drive off and 20 minutes later, we just circle the block. And there's tape and a dude's been shot over there… Our clients care and respect us. They respect us enough to take care of our safety.* – Participant 11

This mutually protective relationship between staff member and client was identified by many participants as a helpful tool to navigate the physical environment and their comfort within it. In this instance, the participant was careful to point out that working with their clients was not what put their physical wellbeing into question, but instead the specific environments surrounding clients. Thus, harm reduction workers may benefit from mechanisms of support that help them navigate not just how to work with their clients but also how to manage interactions with non-clients and uncertainty in the physical environment while doing outreach work in the community.

Additionally, for some participants, physical discomfort from their role extended into their lives outside of work. For instance, a few participants discussed occasionally feeling uncomfortable in their community when returning to a location where they saw something traumatic while on the job. One participant reported feeling that they must restrict their movements when they are not working to avoid areas that might remind them of these traumatic events:

*[T]here is not really a space in [town] I can go without saying, "Okay, I witnessed somebody not make it from an overdose there," or "I remember this gentleman on an overdose here." It becomes that no matter where you go… there is always a negative connotation and memory that's attached to the spaces... [Y]ou're never able to completely detach from that.* – Participant 2

Here, the participant describes how experiences while working can limit one’s movement in the physical environment outside of work. In this way, ensuring that harm reduction staff have sufficient tools to manage such feelings of distress may be a means of supporting staff in ways that potentially transcend work hours.

#### Theme 2: Navigating boundary setting and self-care

Most participants explained how maintaining mental and emotional wellbeing was critical to having longevity in their role and their ability to serve clients. In this context, practicing self-care (e.g., taking time off) and setting boundaries with clients (e.g. turning off work phone after-hours) were identified as protective factors against burnout. Promotion of such boundary setting provides an opportunity for support of harm reduction staff at the individual level.

##### Subtheme 2.1: Feelings of guilt

Unfortunately, many participants asserted that creating such boundaries was very difficult in practice. While most participants recognized the importance of engaging in self-care to improve mental and emotional wellbeing in the midst of a stressful job, many participants explained that protecting one’s own mental wellbeing and comfort was often coupled with feeling guilt or failure toward one’s clientele. One highly experienced participant explained that practicing self-care can feel like one is leaving their clients behind:

*I like to take a week off … But again, I love my job so much that, within that week, I miss my job, I miss my clients, and I'm calling at least one staff member like, “Hey, have you heard from this guy? How's he doing?” And [my teammates are] like “You're on vacation. Stop.” And I'm like, “I can't. I need to make sure he's okay.”*– Participant 13

Another participant explained how they had trouble knowing when to set boundaries, often answering their phone late at night if they felt that their clients needed them:

*How can I feel justified and how can I feel confident or secure in making a decision to not answer their call when I know that they might not have anyone else they feel comfortable calling? That is probably the worst part of my job... I really do want to take care of myself. I really do want to make sure that I'm okay and check in on myself. But then when it feels like that's at direct odds with someone else's well being? That's the hardest thing!* – Participant 16

While staff can be provided with informational support on how to navigate setting boundaries and engaging in self-care, additional support for potential unintended side effects (i.e., guilt) may be necessary as well.

##### Subtheme 2.2: Impact of lived experiences

Supporting the mental wellbeing of harm reduction staff members with lived experience of substance use may be uniquely challenging. For instance, participants with a personal, lived experience of drug use reported that setting boundaries and establishing routines of self-care was crucial to maintaining their own recovery. One such participant reported that though they felt connected to their clients based on shared experiences and community, some client requests were too much for them to reasonably handle:

*I'm definitely working on the “boundaries” thing because I know I come across as like, “I'm going to do anything to help you.” But I also don't want it to interfere with my own sobriety, my children, my home life. There are certain things I can't do.* – Participant 6

Another participant further explained how taxing it can be on one’s health if one is unable to engage in self-care while in a harm reduction role:

*It's amazing to be in recovery or in remission and be able to share your experience, but how do you help [clients] without bringing up your trauma? And no matter how many trainings [I have]... [D]oing this work is still re-traumatizing… [I]t's becoming more painful not taking care of myself. But what does [self-care] look like when this is your lifestyle? I don't clock out. I don't clock out for my life.* – Participant 8

In this way, the inability to “clock out” (i.e., to stop thinking about work) may be more difficult for peer workers and other staff members with shared lived experiences than it is for their participants. Therefore, these staff members may benefit from access to unique, dedicated resources and structures that both champion their distinct impact on harm reduction and help them explore their own reflexivity within the work they do.

### Interpersonal level

#### Theme 3: Establishing and maintaining relationships with clients

Many participants explained that having strong relationships with clients was a key contributor to job satisfaction and feelings of success. For instance, one participant discussed how they had formed close relationships with clients in a way that fueled their continued passion for their work:

*You can share in other people's joys in such a beautiful way – [that] is what has always kept me going… And just to have those moments where… someone feel[s] appreciated and feel[s] safe with you. It's just such a beautiful thing. –* Participant 16

While such experiences are extremely positive, participants explained that sharing in their clients’ accomplishments meant also sharing in their clients’ disappointments. Becoming close to, or even friends with, one’s clients meant that trauma experienced by clients could be felt vicariously by study participants. Some reported that, over time, these feelings made it difficult for them to engage in self-care or create a work-life balance. Similarly, some participants specifically reported that building close relationships with their clients made losing a client extremely difficult to manage while continuing to work. For example, one participant described how it might feel to have clients pass away:

*I right now have maybe seven clients that I've had for over 10 years. And if I was to lose one of them, that would be very hard for me because they've been my client for that long… It doesn't only have to be about family and friends to mourn, we mourn our clients, too.* – Participant 13

Here, this participant compared the relationship with their long-standing clients to that of family and friends. While many participants described this close relationship with clients as a source of pride in their work, some also noted that this closeness led to additional stress when these relationships were cut short (by, for instance, incarceration, death, moving away, etc.). One participant stated that they would want “two weeks of support for a client's death” (Participant 11). In this way, close relationships with one’s clients have the potential to both support the work of staff while also requiring increased support needs upon staff-client relationship strain.

#### Theme 4: Establishing and maintaining relationships with fellow staff

The environments where harm reduction staff are working can be stressful, so many participants pointed to having strong relationships with their colleagues as a critical support in their role. To start, many participants explained the sheer importance of having a partner in the field to lean on and provide safety in numbers. The ability to work as a team and empower each other was essential, especially when navigating variable physical environments or working with new clients. For instance, one overdose responder described how some of their own trauma is revisited through interactions with clients:

*I knew the [client already]. He's volatile, especially towards women… He likes to yell at them. He likes to put his hands on them – and you would not survive. I'm a survivor of domestic violence. I am a survivor of rape… [I]f I feel threatened in any way, I'll tell [my male co-worker], “This one's yours.”* – Participant 7

Here, this participant noted how staff-staff relationships can not only provide feelings of safety and comfort, but also ensure that clients are adequately served. Almost all participants explained how harm reduction staff divide tasks according to their expertise or specific identity in order to best serve the community (e.g., speaking another language to clients, being especially knowledgeable with the transportation system in town, knowing a local doctor to call directly, etc.). This tangible support, facilitated by strong staff-staff relationships, was essential for ensuring participants’ success in their roles and motivating them to continue doing their work.

Furthermore, most participants stressed the importance of having fellow staff members to vent to and debrief with, given the unique stressors one experiences while working. For instance, one participant stated:

*I consider this office – everyone here – to be like family almost. Because if something's going on, they're immediately like “You know what, go home.” Or “You need a second, let's just go for a walk. Let's clear our heads. Let's do something to change the vibe.”... I think that in itself is what helps me to get through the day.* – Participant 4

Unfortunately, harm reduction staff may struggle without supportive staff-staff relationships in the workplace, as some participants noted that tension between fellow staff members can interfere with having a successful working environment. One harm reduction worker described how they felt unsupported by their colleague when they disagreed about how much energy to spend on one client interaction:

And I was already carrying that weight [of working with this client]. And then to have this added weight of, “You care too much.” That's not what I needed from my coworker. I needed them to say ‘D**n, I'm really glad that we met this person and could connect her to something.”… Just to have a moment of pause, that's all I needed. And that's not at all what I got from him. – Participant 16

This interaction shows how staff-staff interactions have the potential to create tension that can interfere with positive work experiences for harm reduction staff (e.g., adding ‘weight’ to already demanding circumstances). Mitigating these types of interactions, through encouraging coworkers to create strong interpersonal relationships, may help foster supportive working environments for staff.

### Organizational level

#### Theme 5: Receiving affirmation and care from supervisors

Many participants noted the importance of receiving direct support and encouragement from their supervisors. For example, one participant described the importance of their boss frequently checking in with them about their life and priorities outside of the workplace:

*So [my boss], actually, is a very big help when it comes to work stress... Because she checks in emotionally. It's not just like, “Oh, what's the job function?”... [but] a boss who's in tune with, obviously, the needs of everybody out there but the needs of us. –* Participant 11

In this way, holistic support from supervisors could protect against work-related stress. A few participants further spoke to the significance of emotional support provided by their supervisors, with one such participant noting, “Sometimes I cry out to my CEO, she doesn't mind wiping my tears” (Participant 14).

Without this kind of socioemotional support, participants ran the risk of growing frustrated with their work environments. One participant shared how they felt frustrated when their ideas and suggestions were not heard or valued by their supervisors:

*One thing that really does frustrate me is when… you meet with superiors and you tell them, “This is not working. This is working and we need to do more of this.”... It's not even heard. And [they] only see data, [they] only see numbers. [They] only want data, [they] only want numbers.* – Participant 13

Other participants described how they felt a similar lack of acknowledgement, explaining that supervisors did not seem to respect their expertise and/or dismissed them when they said they felt uncomfortable or unsafe. In this way, most participants characterized meaningful support from supervisors as more than praise and encouragement and, rather, holistic interest and demonstrated concern in their well-being.

#### Theme 6: Receiving resources and benefits from the organization

Tangible support was also discussed by almost all participants as a key need from their employer. Thus, at this organizational level, participants made clear that the provision of resources and benefits could enable them to perform their job with more confidence.

##### Subtheme 6.1: Training and shadowing

Most participants explained that they received numerous training sessions that helped them comfortably navigate their roles and engage in difficult situations. These training sessions included a breadth of topics such as motivational interviewing, mental health first aid, and safer use. However, participants identified key areas in which they hoped to receive additional training on topics they felt less prepared in, including boundary setting, de-escalation, cultural competency, and navigating resources in the community.

Many participants also wished that new staff members had the opportunity to shadow someone in their position either prior to working as a harm reduction staff or prior to going into the community as a representative of their team, as explained by the participant below:

*I think that it's important for you to shadow in this position before you actually jump in. And maybe [have] some introductions to clients – because at first, they don't trust you and they don't want to come to you, and it takes a while to grow through that.* – Participant 6

Importantly, in the absence of sufficient training, participants described how they independently sought information and training to supplement any gaps in their knowledge in order to avoid not being able to help their clients. One participant noted, “A lot of my training was just me, on my own, figuring things out… It was a lot of teaching myself a lot of things based on lived experiences of other people and the people in the community” (Participant 16). Mitigating the need for self-teaching while on the job may help harm reduction staff feel better supported in and prepared for their roles.

##### Subtheme 6.2: Staffing

Most participants also spoke about the importance of sufficient staffing as a critical organizational resource. Many participants discussed feeling overextended, saying that they were struggling with large caseloads that are difficult to manage. When asked what additional support they needed in their current role, one participant spoke to this, saying:

*The crew we have is fantastic but five or six people can only do so much. When you're serving an entire county, there is only so much we can do… you need feet on the ground and hands in to be able to get it done.* – Participant 5

Though participants noted that they understood why staffing harm reduction roles might be difficult for their organization (e.g., low wages, job-related stress, poor funding), they ultimately noted that this leaves harm reduction staff with too many responsibilities and too little time to manage them.

##### Subtheme 6.3: Compensation and Benefits

Lastly, participants desired other organizational supports such as adequate financial compensation, health insurance, and mental health services. A few participants spoke about having to work multiple jobs in addition to their harm reduction position and their consequent stress. Ultimately, higher wages were identified as a key area participants sought more support. Beyond compensation, participants explained that their mental health would be supported by organizational benefits, including easily accessible mental health services. One such participant described the stress they feel in their role and the consequent need for mental health care:

*I think one critical thing that all harm reductionists need access to is quality therapy and institutional support within whatever organization it is that they work with or whatever collective of people that they are doing this work with to just check in on each other.* – Participant 16

Further noting the trauma stewardship that they undertook in their day-to-day roles, participants called for organizations to provide mental health support such as grief support and paid time off in order to mourn clients’ passing.

### Community level

#### Theme 7: Working with stigmatized population in a stigmatized field

Most participants discussed how stigma toward PWUD and stigma toward the ideology of harm reduction affected their day-to-day roles and often acted as barriers to providing services to the community.

##### Subtheme 7.1: Onus of public education

Some participants described experiences where they were confronted by local community members who disagreed with their work and stigmatized their clients. In response, many participants explained that they felt it was their personal and professional responsibility to educate these community members. One participant described such interactions they had with community members on their mobile van:

*I've had people that have come to the van and they play the role of like, “Hey, can I get some of these syringes? Can I get some of these crack pipes?” And then when I give it to them, they're all like, “You really are gonna give me this? This is my tax dollars paying for this! Do you understand that you're paying people to use drugs?” And if you're not experienced and if you're not trained? Well – this is why training is important – you're gonna get mad, you're gonna get upset, you're gonna think that they're bombarding your van. And it's just a form of staying calm, let them finish, let them yell at you, scream at you... You just educate them.* – Participant 13

In this way, by recommending that other harm reduction workers calmly address these stigmatizing beliefs and educate those that espouse them, this participant is demonstrating how they feel the onus to calmly educate the community is on harm reduction workers, creating an additional burden and stressor in their day-to-day roles.

##### Subtheme 7.2: Ideological misalignment

In addition to facing pushback from community members, some participants explained that they felt pushback toward harm reduction as an ideology from other organizations that also provided services to PWUD. Describing an interaction with an outside organization that had punitively taken a client off of buprenorphine, one participant described this ideological misalignment and tension between organizations:

*[They] like us when we're helping [them] with a client that may not be the easiest to work with, but I don't think they're really aligned with the work that we do… People reach out to us when they want to pass the buck. I mean, I think we get a lot of respect out here, a lot of people admire what we do. But are they aligned with what we do? No.* – Participant 8

In this way, when advocating on clients’ behalf, harm reduction workers are having to navigate ideological tensions with other organizations that might praise harm reduction practices but not actively espouse them. Many participants specifically described this tension when interacting with abstinence-based services in the community.

#### Theme 8: Working within complex and inefficient systems

Also at the community level, many participants described frequent frustrations they felt when helping clients navigate “the system,” describing complicated and onerous experiences they had navigating institutions such as healthcare facilities, mental health services, and housing.

##### Subtheme 8.1: Navigating bureaucracy

Helping one’s client access services around the community became very difficult when bureaucratic barriers limited participants' ability to serve their clients in the ways they want.

For instance, one participant explained how nuanced policies and inclusion criteria often complicated outside referral processes:

*[For] people who want treatment, there are 10,000 [hurdles]. To get them in, you gotta be on this level of methadone or Suboxone, or we don't want you on it at all, you got to pray here, you got to speak Spanish here, you can't have an open wound. It's just too complicated to get somebody actually into a service.* – Participant 11

While participants identified the importance of harm reduction staff members becoming “a bridge” for their clients to negotiate such bureaucratic barriers (Participant 5), harm reduction staff themselves may need support in navigating these systems as well.

##### Subtheme 8.2: Perceived helplessness

Despite many participants stating that they felt pride in helping their clients navigate such complex systems, most participants described how interacting with such systems and bureaucracy led them to feel that their ability to help their clients was stymied. One participant spoke directly to this feeling of perceived helplessness:

*You feel defeated. Because we all come into these roles because we want to be able to help people. And when you know those resources exist out there, but you can't access them, it is maddening. It's so frustrating.* – Participant 1

This experience was echoed by other participants who described how such feelings of helplessness accumulated over time:

*But there's no help for people. Even at the shelters? Money – funding – is just not there. When you have people coming to you desperate, “I know you guys. You guys help everybody, you can help me,” and we can't? I know, for me, it weighs on me. Maybe not the first day, maybe not the second participant, third participant, fourth day. But eventually it's starting to weigh on me. Like, what do I do?* – Participant 8

In this way, while being a central part of harm reduction workers’ roles, having to interface with and navigate “the system” could take a toll on staff mental health and their perceptions of their own capabilities.

## Discussion

This qualitative study aimed to describe and categorize the experiences and perceived support needs of Connecticut-based harm reduction staff. All study participants discussed a variety of ways they currently feel supported and additional ways they hope to be supported in the future. Some common themes emerged across the four SEM levels. At the individual level, harm reduction staff described how they felt their physical and emotional wellbeing could be supported through active boundary setting and self-care, despite working in unpredictable and variable environments. On the interpersonal level, participants’ relationships with clients and relationships with fellow staff members were both revealed as key opportunities for support. At the organizational level, our study revealed that the provision of emotional support and tangible resources was essential to participants feeling appreciated and respected. Lastly, our study indicated that support at the community level might be more difficult for harm reduction workers to achieve, as bureaucratic processes and community-based stigma toward PWUD may interfere with support provision.

Our study furthers existing research on harm reduction workers’ experiences by focusing on the perceived support needs of harm reduction staff in an American context and after the height of the COVID-19 pandemic. With federal funding being allocated to support harm reduction programs for the first time, there is an unprecedented opportunity to bolster harm reduction programs through staff support [31]; these findings identify areas in which this support is most needed. Connecticut can serve as a useful example of the experiences of staff serving different types of programs: those operating primarily within a harm reduction framework, those whose primary objective is getting people into treatment for problematic substance use, and those with an academic research focus. Connecticut is also an exemplar, having been at the forefront of several important harm reduction innovations. It was the first state in which legislative action allowed for syringe exchange to operate (1990) and expand statewide (1992) [32]. It was the first of twelve states that had required prescriptions for pharmacy access to syringes to lift the prohibition (1992) [33]. It was the first state in which a court ruling held that public health took precedence over police enforcement of drug and syringe possession and drug paraphernalia laws (2001) [34]. It was among the first states to pass good Samaritan laws to protect individuals responding to overdoses or providing naloxone to responders from civil or criminal penalties (2011, with protections expanded in 2012,’14, and’15) [35]. It has been a leader in expanding access to medications for opioid use disorder throughout its unitary correction system [36]. Even other parts of the USA that vary significantly in harm reduction service acceptance, legality, and access for PWUD might benefit from the experiences of harm reduction staff in Connecticut. Our study could inform localities that have shown interest in supporting harm reduction work but insignificant experience, resources, or legal support the actions they could take to support staff in their efforts to truly meet people where they are.

Additionally, our analysis was grounded in a SEM framework, which allowed us to explore support needs for stuff on multiple levels. Thus, we can imagine how targeting interventions to specific social-ecological level(s) might improve the provision of support to harm reduction staff. Additionally, use of the SEM provides the opportunity to explore the interplay between levels of influence. In our study, though we focused on the individual, interpersonal, organizational, and community levels, we found that some perceived support needs operated across multiple levels. For instance, relationships between harm reduction staff and their clients exist not only at the interpersonal level, but also impact how staff members feel about their own physical and emotional comfort.

Ultimately, centering the voices of harm reduction staff is critical when evaluating what can best support them. Although participants’ narratives, our study identified an array of unique experiences that pose significant barriers to harm reduction staff feeling supported—experiencing grief from a client’s death, vicarious trauma from client’s adverse events, respondents’ own traumatic experiences, and guilt from setting professional boundaries—they also revealed the many supports for harm reduction staff are already in place in Connecticut-based organizations. For instance, many participants noted they had strong relationships with their manager and had numerous training opportunities related to their roles. Bolstering existing forms of support may be beneficial to improving staff wellbeing and increasing the sustainability of harm reduction programming.

Our study also highlighted key areas that organizations can focus on to address staff burnout and turnover. Overall, organizations should work to ensure there are not only a sufficient number of harm reduction staff members to serve the community, but also that these staff are well-supported. Providing additional training could support staff in ways that help them navigate their roles; for instance, offering training on boundary setting or engaging with non-clientele during community outreach. Additionally, increased compensation for staff members could ensure that staff do not have to take on multiple jobs to support themselves and improve the sustainability of harm reduction programs by ensuring that people are able to establish a career in harm reduction if they choose to do so. Improved benefits, such as access to adequate health insurance and sufficient paid time off, could improve the emotional wellbeing of staff. For instance, therapy and grief support were both identified as role-related benefits that could support participants' self-care and boundary setting (i.e., at the individual level), while also helping staff navigate vicarious trauma or a client’s death (i.e., at the interpersonal level). Participants placed great importance on relationships with fellow staff; creating opportunities for such relationships to form (e.g., creating harm reduction staff support groups, increasing funding for team building, etc.) may help support staff on the interpersonal level. Lastly, organizations should consider how they can continue to advocate for harm reduction across “the system” and within their local communities as a means of reducing the “weight” of harm reduction staffs’ roles. Training staff to advocate for more sustainable funding sources to support staff could include petitioning health insurance providers (most notably Medicaid) to cover harm reduction as healthcare and cover navigators as community healthcare workers and testifying at local meetings and statewide legislative hearings.

### Study limitations

Some key limitations should be noted. First, recruiting currently-employed harm reduction workers could have introduced a selection bias, as harm reduction workers who felt inadequately supported in their roles may have left their positions prior to the recruitment period. Secondly, because our sample exclusively drew from Connecticut-based sites, our findings may not be transferable to other localities, such as states with different harm reduction resource availability or differing regulations around harm reduction (e.g., syringe services, overdose prevention sites, etc.). Thirdly, participants were asked to describe their role in an open-ended fashion which, in hindsight, led to the omission of some potentially important employment-related issues/elements (e.g., number of hours worked per week, etc.). Also, there was key demographic information, such as race and gender, that we decided not to collect, given the relatively limited sample of harm reduction workers in Connecticut. Ultimately, these data collection decisions limited our ability to ascertain differences between workers’ experiences based on these key issues/elements that researchers have previously suggested to be modifying factors.

### Future research directions

While it was not the central research question, our study also expands on the current understanding of how peer workers—with their own lived experiences of substance use—may navigate their roles in ways different than those without this lived experience. For instance, we found that peer workers—with their own drug use history—reported having an especially difficult time creating boundaries due to feeling personally tied to the work and community they serve. Future research should continue to explore the unique support needs of harm reduction workers with lived experiences of substance use.

The programmatic success and sustainability of harm reduction programs will depend upon the wellbeing and job satisfaction of their staff. Harm reduction staff have been found to improve participant engagement in services, provide low-threshold care and fill a gap in the traditional medical system, and create a task-shifting opportunity for specialized staff, such as doctors or emergency medical technicians [37–40]. Our findings provide further evidence that attending to the perceived support needs for harm reduction staff may have an extended benefit for their program’s clients. Future research could aim to understand how common concerns among harm reduction staff may impact burnout and turnover over time. Because these were one-time interviews, we were unable to explore the impact of the "weight" participants felt in their roles over time. Future qualitative research could utilize follow-up interviews to address this knowledge gap. However, provided that almost two-thirds of our sample were in their roles for less than a year, these individuals may be at risk for turnover and burnout if not adequately supported. Future research could explore how the provision of support to harm reduction staff impacts not only staff perceptions of support but also the success of clients accessing harm reduction services.

## Conclusions

Our study suggests that support of harm reduction staff in Connecticut currently exists through some mechanisms but could ultimately be enhanced across multiple levels of the social-ecological model, including the individual, interpersonal, organizational, and community levels. Harnessing the insights garnered from this study provides an opportunity to improve support services for harm reduction staff. Though an end in itself, further research should explore if efforts to support harm reduction staff may also improve outcomes for PWUD.

### Supplementary Information

**Additional file 1**: Harm reduction staff—Interview guide.

## Data Availability

Beyond the excerpts of the transcripts relevant to the study that are available within the paper, full transcripts cannot be shared publicly.

## Abbreviations

PWUD: People who use drugs
SEM: Social-ecological model

## References

[1] Shepard BC (2013). Between harm reduction, loss and wellness: on the occupational hazards of work. Harm Reduct J.

[2] Pike E, Tillson M, Webster JM, Staton M (2019). A mixed-methods assessment of the impact of the opioid epidemic on first responder burnout. Drug Alcohol Depend.

[3] Saunders E, Metcalf SA, Walsh O, Moore SK, Meier A, McLeman B (2019). “You can see those concentric rings going out”: emergency personnel’s experiences treating overdose and perspectives on policy-level responses to the opioid crisis in New Hampshire. Drug Alcohol Depend.

[4] Schulze B (2007). Stigma and mental health professionals: a review of the evidence on an intricate relationship. Int Rev Psychiatry.

[5] Health Policy Initiative, Task Order 1. MEASURING THE DEGREE OF HIV-RELATED STIGMA AND DISCRIMINATION IN HEALTH FACILITIES AND PROVIDERS: WORKING REPORT [Internet]. 2010. Available from: http://www.healthpolicyplus.com/archive/ns/pubs/hpi/Documents/1312_1_Health_Facility_and_Provider_Stigma_Measurement_Tool_.pdf

[6] Olding M, Barker A, McNeil R, Boyd J (2021). Essential work, precarious labour: the need for safer and equitable harm reduction work in the era of COVID-19. Int J Drug Policy.

[7] Mamdani Z, McKenzie S, Pauly B, Cameron F, Conway-Brown J, Edwards D (2021). “Running myself ragged”: stressors faced by peer workers in overdose response settings. Harm Reduct J.

[8] Chen Y, Yuan Y, Reed BG (2023). Experiences of peer work in drug use service settings: a systematic review of qualitative evidence. Int J Drug Policy.

[9] Bardwell G, Fleming T, Collins AB, Boyd J, McNeil R (2019). Addressing intersecting housing and overdose crises in Vancouver, Canada: opportunities and challenges from a tenant-led overdose response intervention in single room occupancy hotels. J Urban Health.

[10] Dechman MK (2015). Peer helpers’ struggles to care for “others” who inject drugs. Int J Drug Policy.

[11] Kolla G, Strike C (2019). ‘It’s too much, I’m getting really tired of it’: overdose response and structural vulnerabilities among harm reduction workers in community settings. Int J Drug Policy.

[12] Kleinman MB, Anvari MS, Bradley VD, Felton JW, Belcher AM, Seitz-Brown CJ (2023). “Sometimes you have to take the person and show them how”: adapting behavioral activation for peer recovery specialist-delivery to improve methadone treatment retention. Subst Abuse Treat Prev Policy.

[13] Pauly B (2021). “It’s an emotional roller coaster… but sometimes it’s fucking awesome”: meaning and motivation of work for peers in overdose response environments in British Columbia. Int J Drug Policy.

[14] Kennedy MC, Boyd J, Mayer S, Collins A, Kerr T, McNeil R (2019). Peer worker involvement in low-threshold supervised consumption facilities in the context of an overdose epidemic in Vancouver. Canada Soc Sci Med.

[15] Greer A, Bungay V, Pauly B, Buxton J (2020). ‘Peer’ work as precarious: a qualitative study of work conditions and experiences of people who use drugs engaged in harm reduction work. Int J Drug Policy.

[16] Wang A, Jawa R, Mackin S, Whynott L, Buchholz C, Childs E (2022). “We were building the plane as we were flying it, and we somehow made it to the other end”: syringe service program staff experiences and well-being during the COVID-19 pandemic. Harm Reduct J.

[17] McNally D. Toronto drop-in network. Bathroom safety & protocol: a practical guide for drop-ins. Available from: https://tdin.ca/res_documents/TDIN%20Bathroom%20Safety%20and%20Protocol%20-%20A%20Practical%20Guide%20for%20Drop-ins.pdf

[18] Hopkins J. Canadian Mental Health Association (CMHA). 2018 [cited 2023 Aug 15]. Reducing Harms: Recognizing and Responding to Opioid Overdoses in Your Organization. Available from: https://ontario.cmha.ca/documents/reducing-harms-recognizing-and-responding-to-opioid-overdoses-in-your-organization/

[19] Gilks T, Hobbs H, Scott A, Gibson E, Amlani A, Buxton J. TAKE HOME NALOXONE: A GUIDE TO PROMOTE STAFF RESILIENCY & PREVENT DISTRESS AFTER AN OVERDOSE REVERSAL [Internet]. 2015. Available from: http://skfn.ca/wp-content/uploads/2020/10/4.-Guide-to-promote-Staff-resiliency-and-prevent-distress-afterreversing-an-overdose.pdf

[20] Wild TC, Koziel J, Anderson-Baron J, Asbridge M, Belle-Isle L, Dell C (2021). Public support for harm reduction: a population survey of Canadian adults. PLoS ONE.

[21] McGinty EE, Barry CL, Stone EM, Niederdeppe J, Kennedy-Hendricks A, Linden S (2018). Public support for safe consumption sites and syringe services programs to combat the opioid epidemic. Prev Med.

[22] Netherland J, Hansen H (2017). White opioids: pharmaceutical race and the war on drugs that wasn’t. BioSocieties.

[23] Martin R, Mann B. Controversial harm reduction strategies appear to slow drug deaths. NPR [Internet]. 2022 Sep 15 [cited 2023 Aug 15]; Available from: https://www.npr.org/2022/09/15/1123108839/controversial-harm-reduction-strategies-appear-to-slow-drug-deaths

[24] Frost MC, Sweek EW, Austin EJ, Corcorran MA, Juarez AM, Frank ND (2022). Program adaptations to provide harm reduction services during the COVID-19 pandemic: a qualitative study of syringe services programs in the U.S. AIDS Behav.

[25] Glick SN, Prohaska SM, LaKosky PA, Juarez AM, Corcorran MA, Des Jarlais DC (2020). The impact of COVID-19 on syringe services programs in the United States. AIDS Behav.

[26] Wenger LD, Kral AH, Bluthenthal RN, Morris T, Ongais L, Lambdin BH (2021). Ingenuity and resiliency of syringe service programs on the front lines of the opioid overdose and COVID-19 crises. Transl Res.

[27] Hennink MM, Kaiser BN, Marconi VC (2017). Code saturation versus meaning saturation: how many interviews are enough?. Qual Health Res.

[28] Braun V, Clarke V (2006). Using thematic analysis in psychology. Qual Res Psychol.

[29] McLeroy KR, Bibeau D, Steckler A, Glanz K (1988). An ecological perspective on health promotion programs. Health Educ Q.

[30] Stokols D (1996). Translating social ecological theory into guidelines for community health promotion. Am J Health Promot.

[31] SAMHSA. SAMHSA Announces Unprecedented $30 Million Harm Reduction Grant Funding Opportunity to Help Address the Nation’s Substance Use and Overdose Epidemic [Internet]. 2021. Available from: https://www.samhsa.gov/newsroom/press-announcements/202112081000#:~:text=This%20funding%20allows%20organizations%20to,programs%2C%20which%20help%20control%20the

[32] Kasprak J. OLR research report: needle exchange programs [Internet]. The connecticut general assembly; 1995. Available from: https://www.cga.ct.gov/PS95/rpt/olr/htm/95-R-1334.htm

[33] New Connecticut Laws to Improve Access to Needles and Syringes: What is Their Impact? [Internet]. Washington (DC): National Research Council (US) and Institute of Medicine (US) Panel on Needle Exchange and Bleach Distribution Programs: Proceedings Workshop on Needle Exchange and Bleach Distribution Programs; 1994. Available from: https://www.ncbi.nlm.nih.gov/books/NBK236646/25144091

[34] Doe V. Bridgeport police department [Internet]. 2001. Available from: https://casetext.com/case/doe-v-bridgeport-police-department-2

[35] Current Laws Related to Opioid Overdose Prevention [Internet]. The Office of Injury and Violence Prevention; 2018. Available from: https://portal.ct.gov/DPH/Health-Education-Management--Surveillance/The-Office-of-Injury-Prevention/Current-Laws-related-to-Opioids-Overdose-Prevention

[36] Department of Correction Expands Medication for Opioid Use Disorder (MOUD) Programs [Internet]. State of Connecticut Department of Correction; 2021. Available from: https://portal.ct.gov/-/media/DOC/Pdf/PressRelease/Press-Releases-2021/DOC-PRESS-RELEASE-re-DOC-Expands-MOUD-programs-061721.pdf

[37] Olding M, Boyd J, Kerr T, McNeil R (2021). “And we just have to keep going”: task shifting and the production of burnout among overdose response workers with lived experience. Soc Sci Med.

[38] Snyder H, Kalmin MM, Moulin A, Campbell A, Goodman-Meza D, Padwa H (2021). Rapid adoption of low-threshold buprenorphine treatment at california emergency departments participating in the CA bridge program. Ann Emerg Med.

[39] Foreman-Mackey A, Bayoumi AM, Miskovic M, Kolla G, Strike C (2019). ‘It’s our safe sanctuary’: experiences of using an unsanctioned overdose prevention site in Toronto. Ontario Int J Drug Policy.

[40] Lennox R, Lamarche L, O’Shea T (2021). Peer support workers as a bridge: a qualitative study exploring the role of peer support workers in the care of people who use drugs during and after hospitalization. Harm Reduct J.

