# Supplementary material for: “It’s starting to weigh on me”: Exploring the Experiences and Support Needs of Harm Reduction Staff in Connecticut using the Social-Ecological Model
Source: Harm Reduct J. 2023 Nov 14;20:168. doi: 10.1186/s12954-023-00898-4 (PMC10644636; doi:10.1186/s12954-023-00898-4)
Supplement: Supplementary file 1 — Additional file 1: Harm reduction staff—Interview guide. [file 12954_2023_898_MOESM1_ESM.docx]

**Appendix 1**

**Harm Reduction Staff - Interview Guide**

**Date:** ___________

**Investigator:** Katherine Hill

**Participant:** _________________

**Role:** __________________

**Employer:** ___________________

**Introduction:** Hi, my name is Katie, and I am a PhD student at Yale University studying public health. My team and I are really interested in better understanding peer navigation for harm reduction and learning from experts in the community to improve our research. Specifically, we want to hear about your experiences, your feelings, your ideas, and your insights into what your clients need and want. To do this, I will ask you a series of questions. Please stop me at any time if you have any questions or concerns, or if you would like to stop or take a break. I expect this interview to take around 45 to 75 minutes to complete.

**ASK:** Are you alright with this interview being recorded for research purposes?

**High Priority Questions**

1. **Participant Characteristics**
   1. Could you tell me your name, and a little bit about yourself?
   2. What organization do you work for, and what is your role within this organization?
      1. Follow up: How long have you been working in this role?
   3. Have you worked in a similar role at another agency prior to your current position? Tell me a little about this.
   4. What brought you to this field? / What is your motivation for doing the work you do?
2. **Training**
   1. Have you received any formal training related to your role?
      1. Follow up, if “yes”: What types of training has your organization offered?
      2. Follow up, if “yes”: In your opinion, what types of training, if any, have been the most helpful to you?
      3. Follow up, if “no”: Have you received formal training outside of your role that you have been able to translate for your work?
   2. What training do you think you might need in addition to the ones you have already received?
   3. If you could change something about how individuals are trained for roles in harm reduction, what would it be?
3. **Skill Set**
   1. In general, how would you describe your approach to being a [peer navigator / community health worker]?
   2. In your opinion, what are the most important skills that a [peer navigator / community health worker] should have when doing their work?
   3. What does a successful day of [peer navigation/being a community health worker] look like to you?
      1. Follow up: Without naming names, can you tell me about any of the attitudes or behaviors of other people that work in your field that might interfere with you having a successful day?
      2. Follow up: Have you learned anything from observing others about what not to do in this role?
   4. How do you maintain trust between yourself and community members/clients?
   5. Are there any additional supports that you need to do the work you do?
4. **Working with Clients**
   1. How would you describe the people you assist?
      1. Follow up: What, if any, are the qualities or characteristics that make it difficult to work with certain people compared to others?
      2. Have you had moments in this role where you feel uncomfortable?
         - Follow up: Can you tell me a bit about the last time you felt uncomfortable?
      3. Follow up: What are the circumstances where you might refuse service to a potential client?
   2. What are your clients' primary concerns out on the street?
      1. Follow up: How do you think your clients come to develop the order of their priorities?
      2. What drives your clients to list certain priorities over others?
5. **Services Provided**
   1. What services do your clients seem to need the most?
      1. Follow up: Consider the full range of needed services – in what ways does your organization make it easier or harder to meet each of these needs?
      2. Follow up: In what ways do the people you serve have the means necessary to improve their health?
   2. What services do you find to be difficult to provide to clients?
   3. Do you feel as though your organization is able to provide quality services to your clients?
      1. Follow up, if “no”: What organizational barriers for clients have been created? / Which of these do you think your organization can remedy?
   4. Are there any services that you feel like your organization should provide but currently do not?
   5. What are the barriers *external* to the organization that limit access to the services they need?
6. **Potential Frustrations**
   1. What gets you most frustrated about your current role during your day-to-day work?
   2. Can you describe the way in which you set certain personal or professional boundaries with the people you serve?
   3. Under what circumstances do you have concerns about your own health or safety when engaged in your work?
   4. What aspects of your professional work do you find especially satisfying? Why?
   5. Do you feel that people in your community respect the work you are doing with your organization?
      1. Follow up: In what ways do you think people not familiar with harm reduction understand the importance of this work?
      2. Follow up: What are the misconceptions about your clients that you think people should be aware of?
   6. This role evidently takes a lot of skill and time and may be stressful at times. How do you prevent yourself from burning out?
7. **Other**
   1. Is there anything else you would like to let me know about your work in this field?

**Closing:** I want to thank you for your time today. Your insight into this field and experiences in the community are so important for researchers like me to understand. I look forward to incorporating your thoughts into our work, and hope we get to talk again soon!

**ASK:** Do you have Venmo or CashApp?
